# Initiation, influence, and impact: adolescents and parents discuss the marketing of gambling products during Australian sporting matches

**DOI:** 10.1186/s12889-016-3610-z

**Published:** 2016-09-13

**Authors:** Hannah Pitt, Samantha L. Thomas, Amy Bestman

**Affiliations:** Centre for Population Health Research, School of Health and Social Development, Faculty of Health, Deakin University, Geelong, Australia

## Abstract

**Background:**

Harmful gambling is a significant public health issue. Alongside the rapid diversification of gambling products, are rapid increases in the marketing for specific types of gambling products, such as online wagering. While concern has been raised about the impact of gambling promotions during sporting matches on the gambling beliefs and behaviours of adolescents, very little research has explored adolescents’ and parents’ attitudes towards the marketing of gambling products within sport.

**Methods:**

A qualitative study was conducted with 59 family groups comprising of at least one parent and one adolescent (14–18 years old) in Victoria, Australia. Parents and adolescents were interviewed separately and asked questions relating to their gambling attitudes and behaviours. They were then brought together, and advertising reception techniques were utilised to prompt discussions about the marketing of gambling during sport. A thematic approach to analysis was used, constantly comparing similarities and differences between and across groups.

**Results:**

Three main themes emerged. First, was *initiation* of sport as a platform for the promotion of gambling. Adolescents perceived that the use of embedded promotions (for example during the match) and the use of athletes in gambling promotions were significant mechanisms for creating an alignment between gambling companies and sporting teams and codes. Second, was the *influence* of marketing messages in creating a perception that gambling was always accessible, and was an integral part of the sporting experience. Third was the *impact* of marketing messages on adolescent’s discourses about sport. Parents described that they had noticed that wagering, and ‘odds’ discussions, had become embedded in adolescents narratives about sporting matches.

**Discussion and conclusions:**

Gambling marketing during sport has significantly increased. While the gambling industry states that it does not aim to intentionally target young people, adolescents are increasingly aware of the relationship between gambling and sport. Future research should explore the impacts and influence of gambling promotions during sport on the gambling attitudes and consumption intentions of adolescents. Effective public health policy is needed to develop comprehensive regulatory frameworks to protect young people from unnecessary exposure to the marketing for this potentially harmful adult product.

## Background

Gambling is increasingly recognised as an important public health problem that may cause significant health and social [1–6] harms for individuals, their families, and communities. Every year, over 400,000 Australian adults experience or are at risk of experiencing harm from gambling [7]. Importantly, for every person that develops harm from gambling products or services, up to ten others are also negatively impacted [7]. Researchers now estimate that the harms associated with gambling are now on a par with other major public health issues, such as alcohol and major depression [8].

While gambling is not traditionally seen as a consumption activity that may pose significant risks for young people (as compared to other similar activities such as alcohol consumption), research suggests that young people are at increased risk of harmful patterns of gambling as compared to adults [9–11]. Australian research shows that about half of all young people will have participated in gambling by 15 years of age, with about three-quarters participating by the age of 19 [12, 13]. Some studies suggest that about 4.0 % of Australian adolescents experience harm from gambling [14, 15], although it is important to note that these studies pre-date the newer forms of gambling, such as online sports wagering. Despite these figures, there is still very limited understanding of young people’s pathways into gambling. Researchers suggest that there may be a range of individual, socio-cultural and environmental factors that may lead to young people’s first experiences with gambling, and may lead some young people to be at increased risk of developing harm with gambling [16]. However, very limited research has explored how gambling industry tactics, such as marketing and the alignment of gambling with culturally valued activities such as sport, may influence young people’s gambling beliefs and consumption intentions [17, 18].

While most gambling products are available in land based environments, concerns have been raised about the growing number of gambling options that are provided via online environments, such as sports based wagering [19]. Wagering is the only form of gambling in Australia to have shown an increase in participation rates in the last decade, and is particularly appealing to young adults. For example in 2014, 10.56 % of 18–24 year olds, and 8.25 % of 25–34 year olds participated in sports and events wagering in the state of Victoria [20]. There may be a number of reasons for this increase in participation, including the ease and 24/7 accessibility of online gambling products, the competitive marketing environment for wagering products on both traditional and social media channels, the lack of a comprehensive regulatory environment for the marketing of wagering products, and the alignment of bookmakers with Australia’s elite sporting codes.

Standard Media Index (SMI) figures from 2011 to 2015 indicate a 160 % increase in advertising spend on gambling (and predominantly sports wagering) in Australia, with $236 million spent on advertising in 2015 [21]. While television advertising for some gambling products (such as Electronic Gambling Machines) is prohibited, there are comparatively very few restrictions relating to the marketing for sports and event wagering in Australia [22]. In 2008, after a High Court ruling in favour of bookmaker Betfair Pty Ltd, registered bookmakers were able to be registered in one part of Australia, while promoting their products in another [22]. Most of Australia’s bookmakers are registered in the Northern Territory, which has caps on taxation, and significantly fewer restrictions on the range of promotional tactics (such as incentives and inducements) that may be used by wagering companies to market their products [23]. While some states and territories prohibit some of these specific promotional strategies, they are still able to be promoted throughout Australia, with ‘fine print’ terms and conditions statements informing customers that the promotion is unavailable in their particular state.

While there is a broad national Australian Commercial Television Code of Practice [24] that details a range of requirements that gambling advertisements need to meet, these requirements have been criticised for not adequately addressing the content of gambling advertisements. For example, while the Code states that gambling advertisements must be socially responsible, must not contain children, must not make gambling appear to be a way of success or achievement, and must contain a statement relating to ‘responsible gambling’ or help services for problem gambling [24], there is very limited specific detail beyond this. There are also few regulations specific to promotions on social media sites such as Facebook and Twitter, with bookmakers regularly using social media sites during matches to provide live commentary based updates on sporting matches, memes, cartoons and funny videos, alongside prompts to bet [25]. Furthermore, there are significant inadequacies relating to the timing of wagering advertising. While the Code stipulates that gambling advertisements are not allowed to be played on television during the ‘watershed’ which replicates children’s viewing hours (4–7 pm), significant loopholes within these regulations means that gambling advertisements are able to be played during the ‘watershed’ if they are within a news, current affairs or sports program [24].

There are also very few restrictions relating to sponsorship relationships between gambling companies and sporting codes. Online bookmakers and casinos have sought to establish alignment with Australia’s elite sporting codes via multi-million dollar sponsorship relationships. While exact monetary figures are rarely disclosed, media reports have suggested a $50 million sponsorship deal between the Australian Football League (AFL) and official gambling partner Crown Bet [26], and a $60 million sponsorship deal between Sportsbet.com.au and the National Rugby League (NRL) [27]. The marketing impact of these sponsorship deals is clearly demonstrated in match based marketing outside of formal television advertisements, including signage around the ground, wagering and casino company logos on match jumpers, and score board advertisements [22, 28]. Sponsorship deals also have an impact on customer sign ups to bookmaker accounts. For example, after a reported $5 million sponsorship deal with the Australian Open tennis tournament, William Hill chief executive James Henderson stated that the partnering had led to *“record customer acquisition rates”* with a reported 1000 customer sign ups per day during the 2 week event [29].

Despite some policy efforts aimed at curbing sports wagering marketing during sporting matches [24], it has been argued that the ‘gamblification’ of sport means it is now almost impossible to avoid the marketing of sports wagering products whilst watching professional sport [22, 30]. Similar to the arguments made about the regulation of tobacco out of sport, researchers argue that there is an ethical tension that exists when sport is heavily marketed as being a ‘family friendly’ activity (and is watched by significant numbers of adolescents) [28], but also contains significant promotions for gambling products that may be ultimately harmful for young people. This is primarily because of the positive associations young people form between products and brands that are associated with sport.

Tobacco control researchers clearly demonstrated that the promotion of tobacco during sport had a significant impact on young peoples “*subconscious positive associations*” between tobacco and sport [31] [pg. 499]. This included young people’s awareness of cigarette brands, and their subsequent consumption preferences for these brands [32]. Similar normalisation trajectories have been shown in young people’s brand awareness of gambling products and companies that sponsor or are advertised during sport. For example, studies suggest that young people demonstrate both brand recall and preference for gambling products that are aligned with sporting teams and codes [33], and state that one of the environments in which they most see marketing for wagering is during sporting matches [18]. A recent study also suggested that young people who are fans of particular sporting codes – in particular the AFL and NRL – may have a higher awareness of wagering promotions than children who follow other sports (such as soccer) [18]. What is less clear from existing research is detailed information about how young people perceive the relationship between gambling and sport. Furthermore, very limited research explores parents’ perceptions of the promotion of gambling within sport.

## Methods

The data derived from this study was part of a larger study investigating Victorian families attitudes towards gambling, including gambling marketing [34]. The broader study investigated perceptions of the causes and consequences of problem gambling, discussions about gambling in social networks, conceptualisations of the risks and benefits of gambling, interpretations of gambling marketing (including lotteries, casinos, horse racing, and wagering), and the effectiveness of mandatory warning messages. For this current paper, we aimed to specifically explore how adolescents and their parents described the processes, influences and impacts of the marketing of gambling within sport on the gambling beliefs and behaviours of young people. To do this, we explored the existing data set utilising three clusters of research questions:

1. How do young people and their parents describe the relationship between gambling and sport? What factors may influence these perceptions?
2. How do young people interpret the messages they see about gambling during sport?
3. Is there evidence to suggest that young people are increasingly viewing sport through a ‘gambling lens’?

### Recruitment of family groups

Family ‘groups’ comprised of one parent and at least one adolescent child (14–18 years old) were recruited to participate in the study. More than one adolescent child was able to participate in the family. Similarly more than one parent was able to participate in the study (although there was not a situation in which more than one parent participated). A commercial market research company was employed to help to recruit the families. The role of the company was to contact the family to assess interest in being involved in the research. If they agreed, they were then contacted by the research team who explained the study in more detail, provided written information, and sought consent from participants. A staged process was used whereby the company was asked to contact families in numerical ‘blocks’. As initial data analysis occurred and greater insight was gained into the data, the company was asked to approach families with more specific characteristics (for example, specific genders, or ages). This is consistent with qualitative research methods, whereby the sample is continuously recruited during data analysis. Written consent was requested and obtained prior to the start of the interview from the parent, and verbal consent was obtained from the adolescent at the beginning of the interview. After the interview, each family group was given a $100 grocery voucher as a reimbursement for the time spent participating in the study. Sampling was stopped when a range of concepts relating to the initial key themes of inquiry were able to be illustrated [35]. Ethical approvals were received from the University Human Research Ethics Committee and the Victorian Department of Justice Ethics Committee.

### Data collection

Interviews were conducted in the family home using a predominantly qualitative data collection method, involving advertising reception techniques to prompt discussion. Interviews lasted between 45 and 120 min (with longer interviews indicative that more than one adolescent in the family had participated). Two researchers attended the interview. This was firstly for safety reasons, but also so that data could be collected in a more efficient way. The process for the interview involved two stages.

First, the parent and adolescent(s) were separated and each completed an interview about their socio-demographic and gambling characteristics; their perceptions of gambling in the community; how they discussed gambling in their social networks; their attitudes towards different forms of gambling, and how they described the risks and benefits of gambling. Parent and adolescent(s) were separated to ensure that there was not undue pressure on adolescents to answer the questions in a certain way. Parents and adolescent(s) were then brought back together to participate in the second half of the study. This utilised visual sociology techniques, whereby examples of gambling promotions from different types of gambling companies (lotteries, casinos, horse racing, and wagering) were shown to the family group to prompt discussions about different types of gambling, and the marketing for these products. Some of these promotions were very overt – for example a bookmaker standing on a professional sporting ground giving the ‘odds’ for a sporting event, through to incidental promotions for gambling, including an advertisement for a casino complex that showed table games as one of a broad range of activities within the casino. Adolescent(s) were asked for their comments before the parents, and we randomised the order that the promotions were shown to avoid an ordering effect [36].

### Data analysis and interpretation

**Fig. 1:**
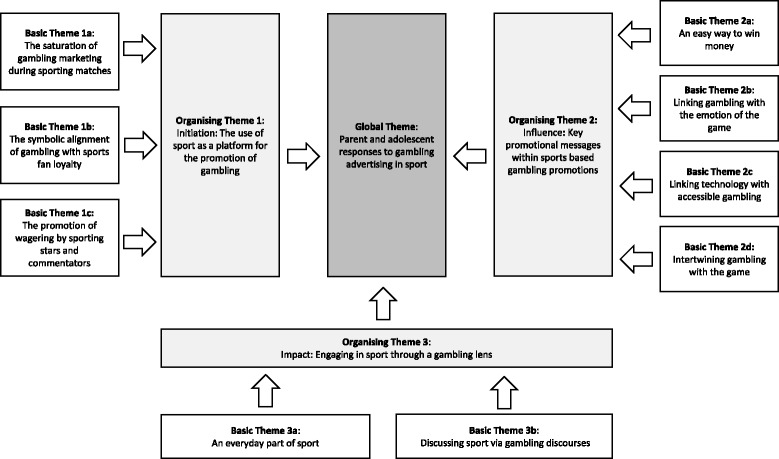
Parent and adolescent responses to the alignment and promotion of gambling within sport

A professional transcription company transcribed audio-recordings of the interviews. Quantitative data were entered into SPSS and analysed using basic descriptive statistics, and QSR NVivo ten was used to manage the qualitative data. The first step in the analysis was to separate the data which related to our key research questions. To do this, HP used *initial coding* to establish analytical ‘fit’ and ‘relevance’ of the data as it related to the research questions. Charmaz [37] describes this process as constructing codes and developing them into categories that “*crystallize participants’ experience*” (fit), and creating an analytical framework that interprets what is happening in the data and “*makes relationships between implicit processes and structures visible*” (relevance) [pg. 54]. HP read the transcripts line-by-line; carefully highlighting the aspects of the interview that related to sports based wagering, the promotion of wagering during sport (hereinafter referred to as sport), or narratives that related to young people viewing sport through a gambling lens. We met regularly as a group, drawing the main themes and subthemes on a whiteboard, discussing how this related to other sections of the interview, to ensure that the context of responses would not be distorted by separating out the data about gambling and sport from the rest of the data. We paid particular attention to the language that was used by participants, and used codes to form the building blocks for our theory. We constantly took notes and discussed these within the team [37], and discussed key differences and similarities between adolescents and parents. We initially compared responses within the parent and adolescent groups, and then compared across the parent and adolescent groups. We looked for similarities and differences in themes. We then used more *focused coding* techniques to group our initial coding categories and explain larger segments of the data (and to form broad themes and subthemes). This process required significant discussion and decision making between the group and we met regularly to discuss and group the distinct concepts and categories within the data. Finally, we progressed to *theoretical coding* in which the key concepts and links within the data were illustrated. Here we developed three initial theoretical codes of *‘initiation’*, *‘influence’*, and *‘impact’*. We then expanded on these theoretical codes by utilising a thematic framework (Fig. 1) initially proposed by Attride-Sterling [38] and developed into a diagrammatic depiction of key themes and subthemes by Thomas and colleagues [39]. This method analyses the data by creating lower level themes or ‘basic’ themes, using these basic themes to form organising themes, and using the organising themes to identify the overall global theme from the data [38].

## Results

### Sample characteristics

A total of 59 parents and 61 adolescents were interviewed across 59 family groups. The sample was distributed relatively evenly across each of the socio economic areas of index (SEIFA) tertiles – low (*n* = 20, 33.9 %), medium (*n* = 16, 27.1 %), and high (*n* = 23, 39.0 %). The majority of parents had completed high school or tertiary education (*n* = 48, 81.4 %). About two-thirds had an income of less than $100,000 per annum (*n* = 38, 64.4 %). Most parents were mothers (*n* = 47, 79.7 %) with a median age of 48 years (*n* = 47.7, range 30–60 years). Just over half of the adolescents in the sample were males (*n* = 33, 54.1 %), with a median age of 16 (*n* = 16.4, range 14–18 years).

### Qualitative analysis

The overall themes and subthemes derived from the data are presented in Fig. 1.

## *Initiation-* the use of sport as a platform for the promotion of gambling

Descriptions from both parents and adolescents indicated that gambling, and more specifically wagering, was becoming embedded within sport. Three main subthemes emerged from the data.

### The saturation of gambling marketing during sporting matches

The first was how gambling was regularly promoted during sporting matches, inherently linking sports and wagering together. Parents perceived that sporting matches were “*saturated*” with advertising and thought that the advertising was “*imposing on your viewing time of the actual event*”. On the whole, parents expressed very negative attitudes about the quantity of gambling, and in particular sports wagering promotions, during sports. Negative descriptions from parents ranged from calling the advertisements “*annoying*”, to more moralistic descriptions, with one participant calling the gambling promotions *“evil”*. Parents described hearing and seeing promotions for sports based wagering on television, the radio, live pop ups during the broadcast, on the scoreboard, and on the big screens at sporting matches. They were particularly concerned how this constant exposure to sports wagering advertising would impact negatively on young peoples’ gambling attitudes and behaviours. For example, the following mother commented:

*“I think anything that’s in your face often enough can be a concern* [for children]*. Constant saturation makes us less conscious of the problems, I feel that we become desensitised to it*.” - 57 year old mother

Adolescents also commented on the extent of gambling marketing they had seen during sporting matches. Many adolescents perceived that there was an inherent association between gambling and sport. This perception was influenced by seeing promotions for gambling (and in particular wagering) “*all the time*” or “*constantly*” during sport, with some adolescents using the word “*everywhere*” to indicate the volume of promotions during sporting matches. Similarly to parents, adolescents had observed the temporal placements of advertisements (for example at half time or breaks in play, and when attending sporting matches) and were also able to recall physical placements of gambling promotions during the match (for example, on signage around the ground and on the big screens at sporting grounds). Most young people referred to the promotion of sports based wagering rather than promotions for other forms of gambling, with some adolescents stating that it was very difficult to avoid being exposed to these promotions during sport. Some stated that the presence of promotions created a natural interest in gambling and in the content of the promotions. For example, the following adolescent stated that although he was not interested in gambling, he was unable to avoid viewing gambling advertisements when he attended football matches:

*“I’m just thinking of the game. Like when I go to the footy, they always have ads and you just sit there and watch them because you’re interested, well not that you’re… but that’s the only thing*.” - 17 year old male adolescent

### The symbolic alignment of gambling with sports fan loyalty

The second subtheme to emerge was the symbolic alignment of gambling, and in particular sports wagering, with concepts of fan and team loyalty. Parents described how the promotional tactics used by sports wagering companies sought to embed sports wagering as part of the emotive aspects of the sporting experience, or as one parent described it, “*the passion of the game*”. Parents perceived that these promotions also tried to encourage wagering as something that “*a typical fan*” would engage in as a natural part of watching a sporting match. A few parents stated that the creatives within advertising particularly targeted sports fans, and used gambling as a vehicle for making fans feel as though they were “*part of the team*” or “*part of the experience*” during a sporting match:

*“If you want to be a part of the team you can be a member* [of that team]*, but if you really want to be part of it you have to bet, and even at the game you can still bet on your phone and things like that*. ” - 41 year old mother

Some adolescents also described how the creatives within some wagering promotions were linked to concepts of fan loyalty. For example, some adolescents interpreted that the creatives in wagering promotions were encouraging individuals to gamble, thus showing support for a particular sports team. Adolescents described how very specific types of gambling promotions, such as logos on team jumpers could create a perception that gambling and the team were aligned with each other. For example, the following adolescent perceived that linking sponsorship with a sporting team helped to “*justify*” gambling and lessened the perceptions of risk associated with the activity. He also commented that gambling logos on jumpers made this relationship between the gambling sponsor and the team more noticeable:

*“I think if you have* [jersey sponsorship] *then you notice it. And then I guess it kind of does justify the idea. Like, if* [the football team is] *backing it then it can’t be that bad.”* - 15 year old male adolescent

However, one adolescent believed that betting would give him a reason to be engaged in the game. He stated that if he bet against his team, and his team lost, that this would still mean that he enjoyed the game:

*“It gives me something to be happy about if my team loses, because I bet against my team. I think that I have a good understanding about soccer to know that I could know the right result. Like an educated guess.”-*17 year old male adolescent

### The promotion of wagering by sporting stars and commentators

The third subtheme relating to the use of sport as a platform for the promotion of gambling was the use of celebrities and athletes to promote gambling. Some parents and adolescents perceived that current and ex-sports stars were used in sports wagering promotions to increase perceptions of trust and the credibility of products. They also perceived that the use of commentary style gambling promotions (whereby an ex-player, gambling representative, or match commentator promoted gambling during a sporting match) was more convincing, trustworthy and authentic than a promotion for gambling that did not use this type of advertising strategy. More parents expressed concern about the use of former athletes or sports commentators in the promotion of sports wagering. Some had strong negative views about this form of promotion, using words such as *“insidious”* and *“destructive”,* and perceived that this was particularly deceptive and influential for young people. This was predominantly because they perceived that young people would trust without question the messages about gambling particularly from current or former athletes, and would not realise that there was a company behind the message. Some stated that this was because young people (and some adults) idolised and respected athletes:

“*…you probably trust the commentator. They are an ex-footballer that you respect…You put more faith in what they say than a politician any day…it’s seamless…To me this is the most insidious, most destructive.”* - 48 year old father

Adolescents also perceived that when the information about wagering ‘odds’ was given by a former athlete or sports commentator, it created a perception that the ‘odds’ of the match were based on the opinions of an expert who had a detailed knowledge of the sport and the match. Some adolescents commented that when messages about sports wagering, and in particular the ‘odds’, came from current or former athletes, they were more likely to trust the messages within the promotions. This is because they were “*legends*” and knew more about sport than “*the average person*”:

*“Because* [ex-players] *normally know everything about sport and maybe even betting…”* - 17 year old male adolescent

Adolescents also described that the appearance of athletes in the promotions for sports wagering created a perception that they were “*connected heavily*” to gambling, and that they “*supported*” or “*were part of it*”. As such, promotions created a perception that athletes were not just promoting the product, but they were also engaged in gambling.

## *Influence* - key promotional messages within sports based gambling promotions

The second key theme from the data were the key messages contained within promotions linking gambling with sport. Four main subthemes emerged from discussions:

### An easy way to win money

The first key message that both parents and adolescents described was the way sports wagering advertising depicted the product as an “*easy way to win*” or to receive financial gain. Parents spoke about the way sports wagering promotions portrayed gambling as easy. Parents were particularly concerned with the way this messaging may impact on adolescent’s perceptions about this activity. For example, some parents thought that the advertising strategies insinuated that “*you’re always going to win”* and would give adolescents a *“false sense that if they gamble they might win”*:

*“I am* [concerned] *because I’m sure there would be lots of kids that would pay attention to the* [advertising] *and who would see it and think it just sounds so easy, a dollar here, a dollar there and you might win big*.”- 45 year old mother

Adolescents said that they thought companies “*made it look like you’re going to win*”, “*say that you can win money*”, “*show how much you can win*” and they “*show people really happy with money*”. For example, one female adolescent when describing the particular creatives for a wagering advertisements, recalled a man saying *“come bet with our company we have the best ‘odds’, you’ll probably win”*. However, some older adolescents were more sceptical about the messages that they recalled within wagering commercials. For example, the following 18 year old stated:

“*Sportbet says to place your bets. They say you’ll never lose. But I don’t think that’s completely true.*” - 18 year old male adolescent

### Linking gambling with the emotion of the game

Overall parents and adolescents perceived that sports wagering promotions suggested that gambling on sport was fun and exciting. Parents commented that gambling promotions which linked gambling with the “*atmosphere*” of the game made gambling appear exciting and could be particularly influential in encouraging individuals to gamble on the match. For example, after viewing an advertisement that showed an AFL team preparing for a match, and leading up to the first bounce of the game, a parent stated that he felt motivated to gamble. This was because of the way in which the advertisement captured the emotion and excitement of the lead up to the game:

*“If an ad was to make me feel like wanting to bet, that one would. Because that feeling of excitement and belonging and glory and going out there on the sporting field type of thing, that buzz, that’s what it really communicated. And I was feeling that buzz, you know it really was a powerful ad*.” - 48 year old father

Adolescents also spoke about the creatives within specific sports wagering advertisements. Some adolescents described that the individuals in the advertisements looked like “*they were having fun*” and others thought that it made “*gambling seem like an exciting thing to do*”. For example, the following adolescent stated that advertisements for sports wagering gave an impression that you would win, and it was a fun activity to engage in:

*“Ads for sports betting tell you to bet with them and make it look like you’re going to win. They make it look positive and fun.”* -15 year old male adolescent

### Linking technology with accessible gambling

The third theme to emerge from the data was that promotions, in particular for sports wagering, made gambling seem accessible and available. Parents and adolescents discussed how sports wagering advertising showcased the availability and accessibility of the product through the promotion of technology. Some parents stated that they were concerned that adolescents were ‘at risk’ from gambling because of the link between marketing for products and the accessibility of these products through mobile technologies and websites. Parents talked about the fact that “*everyone’s got gadgets*” and some of the advertising emphasised that you could gamble from “*the luxury of your own home*”. For example, one mother stated that young people were technologically ‘savvy’ and their understanding and ability to use modern day technology could potentially lead adolescents to new gambling opportunities:

*“…. the new* [gambling service] *you can use on your phone.* [Adolescents] *are phone savvy. They are addicted to that stuff. Now you can bet 24 h a day if you want to. I don’t think it’s good*.” *-* 48 year old mother

Adolescent boys in particular recalled that they had seen marketing that talked about the ease of online gambling, provided incentives to open online accounts, and informed viewers about how to access gambling websites. One boy said they “*show you how to gamble very clearly*”. A few adolescents recalled that advertisements for wagering companies encouraged you to ‘sign up’ to an online account, to *“bet live”* and demonstrated how to access online gambling websites and mobile phone apps by “*saying that they have apps on the phone and they are easy to use and you can use them everywhere”.*

Adolescents also described how they saw people using mobile devices during sports wagering advertisements. This made them feel like they were being encouraged to bet and as though people could bet on their phone, “*anywhere, anytime, anyplace*”. Some adolescents particularly described the link with mobile phone technology. Participants spoke about “*gambling at the footy*” because “*everyone has an iPhone’s these days*” and, “*if you’re at the game, you can just do it.”*

### Intertwining gambling with the game

Finally, participants perceived that gambling promotions encouraged individuals to use gambling as a way of being part of, or more connected to the game. Parents described how sports wagering companies promoted the perception that placing a bet on a favourite team added to ‘*the whole experience of being a football follower*’ and encouraged the mentality that through gambling you become “*part of the game*”. Some parents perceived that this might be problematic for younger fans who may be susceptible to this type of marketing. One parent said:

*“Because they’re right there, so yeah, I think it’s* [a concern]*, I think they’ve tried to intertwine it too much, to make it part of the routine of the game.”* - 44 year old father

Responding to an advertisement that promoted discussion about the sponsorship alignment of sports wagering companies with specific sporting teams, adolescents perceived that wagering sponsorship of a team, and promotions aligned to that team, would ultimately lead people who follow or support that team to want to gamble with the advertised company.

## *Impact* - engaging in sport through a gambling lens

The final theme demonstrated the way adolescents and parents perceived that sport was increasingly viewed through a ‘gambling lens’. There were two subthemes within this theme. The first that gambling was increasingly normalised as an ‘everyday’ part of sport, and the second that gambling discourses had started to influence the way in which individuals, and young people talked about sport.

### An ‘everyday’ part of sport

Parents perceived that gambling was becoming a normalised part of sport. Many attributed this to the excessive promotion of gambling and in particular sports wagering within sport. Some parents thought that this would have a significant impact on the future gambling behaviours and gambling consumption intentions of adolescents. Words such as *“normal”*, and *“normalised”* appeared regularly within parents’ narratives. For example, the following parent commented:

*“It is almost as if they are doing everything possible to normalise gambling and for it to seem such a nice, natural, everyday part of sport. I mean how can you watch a game without having a few dollars on it? That is normalisation.”* - 48 year old father

Other words used by parents described the way in which promotions created an implicit relationship between gambling and sport. Parents used words such as *“subliminal”* and *“brainwashing”* to describe the covert way in which marketing was impacting on adolescents and was contributing to the normalisation of sports wagering during sport. This included a perception that promotions for gambling played during sporting matches, and incorporating sports teams or players, would create positive attitudes towards gambling. One mother described the amount of exposure children may have to sports wagering promotions, and how this may influence young people’s gambling consumption intentions:

“[I’m] *quite concerned. I think children do watch a lot of television, are exposed to a lot of media. They are bombarded by it really. And it becomes more and more normalised. I think especially now that more and more gambling is tied to things that used to be fun, family activities like football and …it’s just seeping in more and more. So that that association between, rather than just going for fun to watching a game, to have that financial interest in it. It’s a social time bomb waiting to happen*.” - 42 year old mother

Although adolescents didn’t specifically use the term ‘normalise’, the way they described sports wagering indicated that they thought that sports wagering was becoming a typical or usual part of sport. For example, adolescents described how the quantity or extent of sports wagering marketing within or before sporting matches could make it seem that gambling was something that everyone did during sport.

There was also some confusion for adolescents about whether a promotion for gambling was a distinct episode of marketing, or whether it was part of the sporting match. For example, when shown a promotional clip of a sports wagering spokesperson on a football field talking about ‘odds’, some adolescents did not believe that this was a promotion, but viewed this as being “*part of the game*” or “*part of the show*”. Some adolescents were unable to understand the persuasive intent of ‘odds’ promotions, and believed that they were there to provide people with information about the game, rather than encouraging them to gamble:

*“I see it more part of the game. It’s information on the game, it’s like anything. They do possession, passes and stuff like that, like how far a player has run. It’s kind of just become part of that*.” - 15 year old male adolescent

Similarly, the following female adolescent believed that ‘odds’ promotions were not encouraging individuals to gamble, but were simply providing information about the sporting match:

*“That’s just giving you the information. That’s all I want. I just want someone to tell me what the ‘odds’ are or the information is, and then if I want to bet I can. They’re not forcing you to bet*.” - 16 year old female adolescent

### Discussing sport via gambling discourses

The second theme related to the way in which gambling discourses had shifted the way young people described and discussed sports. Parents stated that they believed that the promotion of ‘odds’ had changed young peoples’ interactions with sporting matches. For example, parents reported that they noticed that instead of talking about the statistics associated with their favourite teams, or their favourite players, young people talked about the ‘odds’ of sporting matches:

*“I hear kids quoting ‘odds’ now. That’s a concern with advertising that it’s that background filter and it becomes a bit more of a norm to the general population.” -* 47 year old father

Our data analysis also revealed that many adolescents discussed the term ‘odds’ during the interview. Some adolescents said that they discussed the “*sporting ‘odds’, and what* [sic] *team are at what* [sic] *‘odds’*” when talking with their friends and/or family. Others recalled that ‘odds’ were promoted in a variety of different ways, including the display of changing ‘odds’ during sporting matches, and whether or not a company had *“the best odds”* for a particular event. Adolescents were also able to describe what they thought the ‘odds’ meant. Most adolescents interpreted ‘odds’ as an indicator of whether a team was likely to win: “*If they’re* [sporting team] *going to win or not*” or “*If a team is more likely to win then the ‘odds’ would be lower because there’s a greater chance that they’re not going to give away as much money*”.

## Discussion and implications for public health

This exploratory study provides information about the range of factors that may influence how young people may interpret and apply the messages that they see about gambling during sport. The study aimed to understand: 1) How adolescents and their parents describe the relationship between gambling and sport, and the factors that may influence these perceptions; 2) How adolescents interpret the messages they see about gambling during sport; and 3) Whether there is evidence to suggest that adolescents are starting to view sport through a ‘gambling lens’.

The findings from this study raise a number of points for discussion.

First, adolescents and parents in this study were aware of the increasing alignment of gambling and sport. Most commonly, this perception was related to the promotion of gambling products within sporting matches. While a range of gambling products and services are promoted during sporting matches – including casinos, keno, and lotteries – the type of gambling that was referred to the most by participants in this study were promotions for sports wagering. This is perhaps unsurprising given that researchers have highlighted the saturation of promotions for these products in sporting matches – both during traditional commercial break advertising, and embedded within match play via promotions such as logos on jumpers, signage at the ground, pop ups, and tickers [22, 28]. Studies have also shown that children are able to recall and link sports wagering and gambling companies with specific sporting teams and codes [17, 33], have a high unprompted recall of gambling brands [17, 18], and can recall and describe seeing advertisements during sport for specific sports wagering companies [18]. This study contributes to these previous studies by demonstrating that adolescents have an awareness of promotions outside of traditional commercial break advertising, that they are able to describe the content of these specific promotional tactics, as well as the timing or placement of these promotions during sporting matches. Furthermore, the study demonstrates that adolescents perceived that the use of current and ex-athletes in either the implicit or implied endorsement of gambling products is a particularly influential tactic in aligning gambling with sport. Research from other public health issues has shown the influence that celebrities and athletes can have on adolescent’s uptake of behaviours and attitudes towards harmful products [40, 41]. Further research should explore whether the endorsement of gambling products by current or ex-athletes plays a role in positively shaping young peoples’ gambling beliefs, brand preferences and consumption intentions.

Second, while gambling companies repeatedly argue that adolescents are not the target of their promotions [42], and that parents should be responsible for educating their children about gambling, this study suggests that parents are increasingly concerned about the excessive promotion of, in particular, wagering advertising in sport. Our findings suggest that parents may feel that they are unable to counter the persuasiveness and volume of promotions for gambling, particularly when they are aligned with concepts associated with supporting your team or fan loyalty. While those involved in the promotion of gambling products in sport (including sporting codes, broadcasters and the gambling industry) have done little to address these concerns, public health practitioners should aim to work with parents to ensure that their opinions and concerns are regularly heard by these agencies, and to advocate for change [43]. Further research should more comprehensively investigate the range of possible regulatory responses to gambling advertising within sports-based programming to ensure that this type of marketing does not negatively influence vulnerable populations. While the most obvious way forward is to close regulatory loopholes, which allow gambling to be advertised within children’s viewing hours if they are within sporting matches [22], this study suggests that policy makers should consider how they expand regulatory frameworks to encompass a wider range of promotions that may occur outside of traditional commercial break advertising. One idea, as suggested in limiting the exposure of alcohol marketing to children, may be to develop an audience threshold for promotions, whereby gambling promotions are banned or significantly restricted if the number of young people in an audience is over a certain number or percentage of the viewing audience [44]. It is important that these initiatives are developed independently of those companies or organisations that may have a commercial interest in the promotion of gambling products. Tobacco control also provides an important historical template for public health policy makers to follow. By 1976 direct tobacco advertising was banned in Australia, with further bans occurring during the 1990’s to restrict the sponsorship of sport by the tobacco industry [45]. It is widely regarded that this approach, as part of a comprehensive approach specifically targeting all advertising mechanisms, contributed to a decrease in the uptake and prevalence rates for smoking [45, 46].

Third are the messages that adolescents interpret from the gambling promotions they see within sport. Adolescents in this study interpreted the messages about sports wagering, as being easy, accessible, and fun. Research from other gambling product advertising (such as lotteries) have also shown that the messages of winning and that gambling is easy, are recalled and viewed positively by children and adolescents [47, 48]. Derevensky and colleagues (2010) described that these types of promotions are influential in young people’s reasons for wanting to engage in gambling [48]. While our study did not specifically explore the impact of these promotions on consumption intentions, this is an important area for future consideration. This should include understanding how promotions that highlight the role of technology may influence gambling consumption intentions in young people.

Finally, parents were particularly concerned about promotions that implied that gambling was part of supporting a team. Marketing techniques, which aim to embed sports wagering as a ritual within sport may have an influence on children’s ability to recall gambling brands and their brand preference [17, 18, 33]. While there is an assumption that adolescents are generally able to understand the persuasive intent of marketing (in this case that marketing for gambling is encouraging individuals to gamble) [49], adolescents struggle to identify ‘odds’ announcements as marketing techniques used to encourage gambling, with some seeing it as information about the potential outcome of a game. This raises questions about whether discourses from the gambling industry which seek to embed gambling as an inherent part of sport, are shaping young people’s attitudes towards gambling. Language is a factor that plays a significant role in the shifting of social norms, particularly relating to the consumption of unhealthy products [50, 51]. There were some indications in this study that young people’s discourses about sport increasingly involve discussions about gambling, and in particular gambling ‘odds’. Further research should explore the range of mechanisms that may be contributing to a normalisation trajectory of gambling as an inherent and accepted part of the sporting experience, and whether some groups of young people may be particularly influenced by specific mechanisms.

It is important to acknowledge the study limitations. Firstly, this is an exploratory study, and the study findings cannot claim to represent the attitudes and opinions of all adolescents who watch sport and their parents. The study did not aim to provide a representative sample of all Australian households, but to generate a range of attitudes and opinions about gambling. A further limitation of this study was that the parental sample was skewed towards mothers. Given that men more frequently participate in gambling on sport, it may be that a larger sample of fathers may have yielded different results. The exclusion of non-English speaking participants is also a limitation. Finally, given that this is a new area of discussion for many young people and their parents, we used advertising materials to prompt discussion about some very specific forms of marketing, such as ‘odds’ announcements. Future research should also seek to examine unprompted or implicit awareness and interpretation of these types of promotions.

## Conclusions

The findings from this study provide an important starting point for more comprehensive investigations about the impact and influence of gambling marketing during sporting matches on adolescents. This study suggests that there may be a process whereby marketing is used to *initiate* a link between gambling products and sport, *influence* the belief that gambling is an integral part of sport, and create an *impact* on the use of gambling discourses to discuss and describe sport. We would conclude that these processes may be shaping a normalisation trajectory in which adolescents believe that gambling is a normal and valued consumption activity during sport, which may in turn influence their consumption intentions. As with other areas of public health, effective public health policies and advocacy strategies will form an important part of a comprehensive public health response to gambling harm. Engaging young people and their parents in developing solutions will be an important part of this response. Public health practitioners have many historical templates from other areas of public health (such as tobacco control) with which to develop comprehensive research and policy responses to gambling industry marketing strategies.

## Abbreviations

AFL: Australian football league
NRL: National rugby league
SEIFA: Socio economic areas for index
